# Report of Additional Cases of Febris Intermittens, Treated with the Extract Pinckneya Pubens

**Published:** 1864-09

**Authors:** A. M. Fauntleroy

**Affiliations:** Medical Director, Wilmington.


ART. IV.—*Report of Additional Cases of Febris Intermittens, Treated with the Extract Pinckneya Pubens.* By A. M. FAUNTLEROY, Medical Director, Wilmington.

Case 1.—L. B. Bynum, private company "A," 2d regiment engineers. Patient has had repeated attacks of fever since September, 1863. Form of fever, "Tertiana," hour of expected attack 4 o'clock, May 25th, 1864, P. M. 7 A. M., pulse 68—6 grains administered, and the same ordered every hour. 12 M., pulse 80—slight moisture of surface of body; patient comfortable; complained of no ill effects from the medicine. 1 P. M., pulse 80—diaphoresis the same, patient quiet; 6 grains given. 2 P. M., 6 grains given. 3 P. M., 10 grains given; pulse 78—diaphoresis profuse; patient restless, but complains only of heat. 3½ P. M., 10 grains given; pulse 76—diaphoresis very profuse. The paroxysm did not appear. During the night the patient had two very copious, dark evacuations from the bowels. He improved in every respect; his appetite increased in an incredible manner. He remained *well,* attending to his ordinary duties, until about ten days ago, (5th of July) when he was attacked with bilious remittent fever, from which he is now rapidly convalescing. 68 grains in all were administered.

Case 2.—Spy, private company "B," 10th North Carolina battalion of artillery. Patient strong and robust; has had five attacks of intermittent fever; quotidiana form; hour of expected chill, 11 o'clock, June 5th. At 6 A. M., 6 grains were given according to directions given the evening before. 7 A. M., pulse 64—6 grains given, and the same ordered at 8 and 9 o'clock. 10 A. M., pulse 85—very slight moisture on surface of body. 11 A. M., pulse 92—10 grains given; no moisture; patient complained that the chill was coming on. 11½ A. M.; chill came on at 11.25 minutes, but slight. 12 M.; chill has passed off; fever succeeded; pulse 110. The fever lasted two hours and a half, followed by the usual degree of diaphoresis. June 6th. Patient reports himself as "right smart better;" pulse 72. 6 A. M., 6 grains given, and the same ordered every hour, which had the effect of breaking the chill and fever; diaphoresis began at 10 o'clock. The patient returned to duty two days after.

Case 3.— —— Poole, private company "A.," 2d regiment engineers. Has had occasional attacks of intermittent fever throughout the winter; form Tertiana; time of expected chill, 10 o'clock P. M., June 9th. 7 A. M., pulse 75—rather weak; 10 grains given. 8 A. M., pulse 80—patient comfortable; 6 grains given. 9 A. M., pulse 90—patient restless; complained of pain in back. As the pulse had risen so rapidly, 3 grains only were given at 10 o'clock. 10 o'clock A. M., chill came on two hours earlier than anticipated; both chill and fever, however, were of shorter duration than usual. June 10th. The pinckneya pubens was administered in 6 grain doses every second hour during this day. June 11th. 6 grain doses were given every hour, commencing at 6 o'clock A. M., and had the effect of warding off the chill; diaphoresis, as usual, supervened.

From a careful study of the cases herein reported, I am forced to adhere to my views rendered in the report of the 22d of April, 1864, to wit: That the extract has undoubted antiperiodic properties, still it is too slow in its action to be used as a substitute for quinia sulphas. It has, with one exception, always produced diaphoresis. Its therapeutical action is principally that of a tonic, and it deserves a position in the front rank of vegetable tonics. From the tardiness of its action, and its effect upon the vascular system, together with its manifest invigoration of the digestive organs, I am induced to think its energy as an agent is displayed through the organic nervous system.

